# Distribution of ABO and D antigen expression in Yogyakarta, Java Island: a pioneer large-scale study in Indonesia

**DOI:** 10.1186/s13104-024-06914-5

**Published:** 2024-09-18

**Authors:** Hasna Fadlilatul Bidayah, Teguh Triyono, Yann Fichou, Rarastoeti Pratiwi, Diah Nurpratami, Abdul Salam Sofro

**Affiliations:** 1 Biotechnology Study Program, Graduate School, Universitas Gadjah Mada, Yogyakarta, Indonesia; 2 Department of Clinical Pathology and Laboratory Medicine, Faculty of Medicine Public Health and Nursing, Universitas Gadjah Mada, Yogyakarta, Indonesia; 3 Dr Sardjito General Hospital, Yogyakarta, Indonesia; 4 Univ Brest, Inserm, EFS, UMR1078, GGB, Brest, France; 5 Laboratory of Excellence GR-Ex, Paris, France; 6 Laboratory Biochemistry, Faculty of Biology, Universitas Gadjah Mada, Yogyakarta, Indonesia; 7 Politeknik Kesehatan Bhakti Setya, Yogyakarta, Indonesia; 8 Indonesian Red Cross, Yogyakarta, Indonesia

**Keywords:** ABO, Rh, Blood Group System, Distribution, Indonesia

## Abstract

**Objective:**

Here, we sought to report ABO and D antigen distribution in blood donors from Yogyakarta, Java Island, Indonesia. Phenotype data (ABO/D) from donors who donated blood between January 1, 2018, and December 31, 2023, at the Yogyakarta Blood Donor Unit were extracted from the blood donor registry, and phenotype frequency was calculated subsequently.

**Results:**

In the 245,307 blood donors collected over six years, ABO phenotype frequency: O (frequency: 38.5%) > B (29.4%) > A (24.1%) > AB (8.0%). The D-positive phenotype was far more common (99.5%) than the D-negative phenotype (0.5%). The phenotypic pattern globally is similar to previous reports in Southeast Asia. The D antigen distribution is similar to world distribution as the most common blood group. For the first time in Indonesia, this distribution of ABO and D phenotype is reported in a large-scale study. This work is a pioneer in the coordinated optimization of transfusion guidelines at the national level.

## Introduction

Among the 45 blood group systems officially reported to date by the International Society of Blood Transfusion, [1] ABO (ISBT 001) and Rh (ISBT 004) are the most clinically relevant. Indeed, the antibodies directed against these antigens can potentially trigger a hemolytic reaction in patients [2–5]. ABO blood group antibodies may be responsible for severe hemolytic transfusion reactions (HTRs) in patients transfused with incompatible red blood cells (RBCs), [6] while the hemolytic disease of the fetus and newborn (HDFN) due to ABO antibodies, although relatively common, is usually of milder clinical significance. [7, 8] The D antigen harbored by RhD transmembrane protein is the most immunogenic, among Rh antigens. D-positive (D+) RBCs from donors and fetus(s) can elicit alloanti-D production in D-negative (D–) patients and pregnant women, respectively, a mechanism known as alloimmunization. [9] In alloimmunized patients, a novel exposure to the D antigen may result respectively in HTR and HDFN with a broad range of severity ranging from asymptomatic to fatal outcomes. [2]

In transfusion medicine and obstetrics, prevention of alloimmunization and potential subsequent complications, especially in cases of chronic transfusion, typically relies on blood group typing to ensure compatibility between donors and patients and/or to reinforce monitoring in pregnant women when necessary. [3, 4, 10] Since the discovery of ABO and Rh system, numerous studies available in the literature have reported the distribution of the main antigens, including A, B, and D, in various populations. From these reports, it has long been known that antigen frequency, directly driven by blood group gene polymorphism, varies considerably as a function of the origin of the population of interest. Therefore, knowledge of this distribution can help manage blood banking efficiently at the local, regional, and national levels.

Indonesia is a vast country in Southeast Asia composed of thousands of islands and accounts for the fourth most populous country in the world, with a population estimated to be > 280 million inhabitants, including various ethnicities. [11] To our knowledge, blood group antigen frequency in Indonesia is barely available in the literature and remains to be further documented throughout the country. Thus we thought to report for the first time the frequency of ABO and D antigen expression in Yogyakarta, Java Island.

## Main text

### Materials and methods

Blood group records (i.e., ABO/D phenotype) from the blood donors’ database of the Yogyakarta Blood Donor Unit between 1st January 2018 and 31st December 2023 were collected retrospectively. Blood donors were selected strictly according to the nationally standardized selection criteria established by the blood bank. Blood sample was taken from male and female volunteer blood donors aged 18–65, weighing more than 45 kg with normal hemoglobin values. Routine ABO and Rh serotyping in donors was performed with automated microplate and microfiltration systems with E.M. Technology (Qwalys 3, Diagast, Loss, France) with the following reagents: ABDLys, Magnelys, Bromeline, Hemalys 1 A1B (Diagast, Loos, France). Manual tube tests were performed with Eryscreen anti-A, anti-B, and anti-D (Tulip Diagnostics, Goa, India). Subgrouping and other blood group antigen tests were not performed. Only donors with concordant forward and reverse typing results were selected for data analysis. Data were processed using a Microsoft Excel datasheet, and phenotype frequency was calculated.

## Results

Over six years, a total of 245,307 donor blood samples (average: 40,885 donors/year) were analyzed to determine ABO and D blood group antigen distribution. The ABO and D antigen distribution occurred in the following order O + > B+ > A + > AB + > AB−> O− > A− = B- (38.42% > 29.35% > 24.10% > 7.67% > 0.31% > 0.08% > 0.04% & 0.04%) (Table 1). In the ABO system, the most common phenotype was O (38.5%) followed respectively by B (29.4%), A (24.1%), and AB (8.0%). In the Rh system, the frequency of D + donors is dramatically high (99.5%), while D– donors account for only a minor subset (0.5%) (Table 2).

**Table 1:** Phenotypic distribution of ABO and D antigens in blood donors from Yogyakarta (Central Java Island, Indonesia) between 2018 and 2023

Phenotype		2018			2019			2020			2021			2022			2023			Total	
		*N*	%		*N*	%		*N*	%		*N*	%		*N*	%		*N*	%		*N*	%
O+		17,589	**38.43**		18,476	**38.65**		13,328	**38.97**		13,097	**38.93**		14,375	**37.97**		17,374	**37.74**		94,239	**38.42**
O-		26	**0.06**		52	**0.11**		18	**0.05**		26	**0.08**		30	**0.08**		32	**0.07**		184	**0.08**
B+		13,529	**29.56**		14,054	**29.40**		9,807	**28.68**		9,848	**29.27**		11,137	**29.41**		13,628	**29.60**		72,003	**29.35**
B-		31	**0.07**		25	**0.05**		12	**0.04**		7	**0.02**		5	**0.01**		11	**0**,**02**		91	**0.04**
A+		11,085	**24.22**		11,545	**24.15**		8,288	**24.23**		7,987	**23.74**		9,084	**23.99**		11,123	**24.16**		59,112	**24.10**
A-		21	**0.05**		38	**0.08**		11	**0.03**		5	**0.01**		17	**0.04**		15	**0.03**		107	**0.04**
AB+		3,470	**7.58**		3,597	**7.52**		2,716	**7.94**		2,617	**7.78**		2,911	**7.69**		3,503	**7.61**		18,814	**7.67**
AB-		18	**0.04**		15	**0.03**		19	**0.06**		53	**0.16**		303	**0.80**		349	**0.76**		757	**0.31**
Subtotal/year		45,769	**100.00**		47,802	**100.00**		34,199	**100.00**		33,640	**100.00**		37,862	**100.00**		46,035	**100.00**		245,307	**100.00**

*N*: number of blood donors

**Table 2:** Frequency of ABO and rh D blood groups in Southeast Asia, Asia, and other countries

	ABO (frequency, %)					Rh (frequency, %)			*N*	Reference
Region/country	O	A	B	AB		D+	D–			
South East Asia										
**Indonesia**	**38.5**	**24.1**	**29.4**	**8.0**		**99.5**	**0.5**		**245**,**307**	This study
Laos	37.7	19.8	35.6	6.9		100.0	0.0		464	^16^
Malaysia	39.2	24.7	30.3	5.8		99.3	0.7		760	^17^
Myanmar	36.5	25.2	33.3	5.0		–	–		222	^15^
Philippines	45.4	24.0	24.9	5.7		98.9	1.1		5,953	^12^
Thailand	37.7	21.4	33.6	7.3		99.7	0.3		1,382,980	^14^
Vietnam	49.9	21.8	25.0	3.3		100.0	0.0		423	^13^
Asia										
India	37.12	22.88	32.26	7.74		94.61	5.39		9,686	^19^
Pakistan-North_Western	28.79	28.16	32.34	10.71		90.72	9.28		226,963	^21^
Pakistan -Faisalabad	32.78	22.58	29.79	14.83		81.01	18.99		17,205	^20^
Pakistan-Safdarabad	33.74	25.28	33.81	7.15		90.00	10.00		13,477	^20^
China	34.20	28.72	28.17	8.91		-	-		23,697,367	^29^
Japan	29.25	38.65	22.15	9.95		-	-		4,464,349	^28^
Others										
Australia-nonAborigin	45.2	37.1	13.0	4.7		83.7	16.3		2,657	^30^
Australia-Aborigin	56.6	39.7	2.9	0.8		97.6	2.4		1,686	^30^
Antananarivo	41.60	22.61	29.66	6.13		98.9	1.1		45,857	^23^
Burkina Faso	43.30	22.54	28.56	5.60		92.24	7.76		81,486	^18^
Germany-SouthWestern	41.21	43.26	10.71	4.82		-	-		62,161	^26^
Guinea	48.88	22.54	23.86	4.72		95.94	4.06		59,452	^22^
Mexico	61.82	27.44	8.93	1.81		95.58	4.42		271,164	^27^
Uganda	50.3	24.6	20.7	4.5		97.97	2.03		23,504	^25^
United States	46.6	37.1	12.2	4.1		85.4	14.6		3,086,215	^24^

*N*: number of individuals analysed in the respective studies; –: not reported

## Discussion

In this study, the distribution of ABO and Rh blood group systems in donors of a blood bank directory from Yogyakarta, Java Island, was reported retrospectively over six years (2018–2023). To our knowledge, this is the first study conducted on such a large scale in Indonesia.

In this research, the distribution of the ABO blood group phenotype is O > B > A > AB, which is a pattern typically shared by several South East Asian countries, [12–17] and several other countries (Table 2). [18–23] However, variability may be observed between countries. Still, the world distributions occur in the following order: O > A > B > AB [5, 24–30]. The blood group A was more dominant in the Northern Hemisphere. [5] The blood group distribution pattern was complex, clinal, and discontinuous. The distribution pattern can remain constant for several decades, [31] but also can change over time. [32]

The D antigen was found to be carried by the vast majority of donors (Table 2: 99.5%), which is also a general observation in East and Southeast Asia. It is worth mentioning that the D antigen frequency reported here in donors may not reflect precisely the actual distribution in the general population. Indeed, due to a shortage in the availability of D– RBC units in Indonesia, D– donors are encouraged to give their blood regularly. Thus, the frequency of D– individuals may be somewhat overestimated in this study. Next, it will be essential to investigate the nature of D– donors by additional serological and molecular methods to address the potential Asian-type DEL samples in this subset, which have recently been recommended to be considered as D + donors and patients, conversely to the true D– individuals. [33]

Because it is well known that blood group antigen expression varies between populations due to specific genetic variations, knowledge of the distribution of blood groups at a local level/regional/national is critical for blood banks, transfusion services, and patients’ healthcare. This report describing the distribution of ABO and D phenotypes among donors in Indonesia is thought to be a milestone towards the establishment of general guidelines for blood group distribution in the country. Our findings will be helpful to clinicians in their daily practice, planners, and policymakers for optimizing the management of RBC stocks, as well as non-governmental organizations involved in blood supply. This report also has valuable implications for modifying and producing blood products (blood components) to prevent shortages. This finding implies that blood type O is the most available blood group and is more advantageous for the population in transfusion practices. Blood group O, especially O-negative, is crucial to transfusion practice. In Asian countries, including Indonesia (Yogyakarta), the frequency of the O-negative phenotype was lower than in European countries so, the regional bank needs to improve O-negative blood availability to avoid shortages. [18]

Finally, Indonesia is a large country known for its complex geography and heterogeneity in terms of ethnicity, thus assuming a broad range of genetic backgrounds. While we are currently involved in another comprehensive study nationwide, we encourage other blood banks nationwide to publish their data to increase the amount of data and get a global overview of blood group distribution. Indonesian policymakers need to enhance the standard for blood grouping tests, which include subgrouping and other blood group antigens. While these tests may not be available in regional blood banks, having them accessible in the national blood reference laboratory would be beneficial.

## Limitations

Our study has a few limitations that need to be acknowledged. Our study uses a relatively small sample size compared to the Indonesian population. Due to our testing reagent limitations, there was no subgrouping test for routine serotyping. Serotyping for forward grouping was performed with anti-A, anti-B, and anti-D, and reverse grouping was performed with A1 and B cells. Testing for weak D and H lectins was not performed. Also, in this study, D– donors only donate their blood with a special invitation, so D– distribution may be higher. We are still conducting a more comprehensive nationwide study involving blood donors from different regions and backgrounds in Indonesia to confirm these findings and identify any region-specific variations in the ABO and D antigens among blood donors, considering the country’s heterogeneous geography, ethnicity, and genetic variation.

## Data Availability

The datasets used during the current study are available from the corresponding author upon reasonable request.
